# Supplementary material for: Are carbon emissions trading and green financial instruments synergistic? -Comprehensive quantitative research based on content analysis
Source: PLoS One. 2024 Mar 7;19(3):e0298601. doi: 10.1371/journal.pone.0298601 (PMC10919601; doi:10.1371/journal.pone.0298601)
Supplement: S1 Data — (ZIP) [file pone.0298601.s001.zip › Data/地级市Z_F工作报告.zip!/╣≤╓▌/░▓╦│╩╨/└┤╫╘╣1⁄2╓┌║┼í╛┬φ┐╦╩2╛▌═°í┐_2013.docx]

各位代表：

　　现在，我代表市人民政府向大会作工作报告，请予审议，并请市政协委员和其他列席同志提出意见。

　　一、2012年的工作回顾

　　2012年，在省委、省政府和市委的领导下，在市人大、市政协的监督、支持下，市人民政府坚持以邓小平理论、“三个代表”重要思想、科学发展观为指导，紧紧围绕市第三次党代会和三届人大一次会议确定的目标任务，团结和依靠全市各族人民，扎实苦干，攻坚克难，全力以赴推进工业化、城镇化、农业现代化和旅游产业化，主要经济指标实现增比进位，城乡面貌明显改变，人民生活持续改善，社会事业全面进步，全市经济社会发展呈现出提速赶超的良好态势。

　　全年实现生产总值352.62亿元，增长15.4%；全社会固定资产投资400.06亿元，增长64.4%；财政总收入87.40亿元，增长32.9%，公共财政预算收入37.50亿元，增长41.5%；社会消费品零售总额97.53亿元，增长16.2%；城镇居民人均可支配收入18617元，增长14.2%；农民人均纯收入5088元，增长16.5%；金融机构存款余额483.14亿元，贷款余额350.23亿元，分别比年初增长12.9%和30.4%。三次产业结构从2011年的15.6∶39.4∶45.0调整为15.1∶38.9∶46.0。在全省经济发展增比进位综合测评排位中，我市从2011年全省挂末上升到第六位，所有县区都进入前50名。

　　——我们全力加大跑上跑外力度，支持政策进一步落实。全市上下抢抓国发〔2012〕2号和黔党发〔2012〕15号文件出台的重大机遇，加大“进京跑部”、“进厅入处”力度。73个省直部门先后出台支持安顺加快发展的具体政策措施。全年共获得中央和省转移支付资金97.06亿元，比2011年增加17.52亿元，增长22%。一批重点项目建设加快推进，安电三期获得国家“路条”，马马崖水电站通过国家核准截流验收。许多多年来想办但一直没有办成的大事难事，取得重大进展。

　　——我们全力扩大固定资产投资，发展基础进一步夯实。全市50万元以上固定资产投资完成298.16亿元，增长80.1%，增速排名全省第一。完成交通建设投资68.6亿元，长昆快速铁路安顺段建设稳步推进，安普高速公路、惠兴高速公路惠水至镇宁段建成通车，新增高速公路通车里程106.9公里，建成通村油路（水泥路）607.7公里。完成水利建设投资11.3亿元，黔中水利枢纽、“引千入虹”等重点水利工程建设进展顺利，石朱桥、新场河水库开工建设，黄家湾水利枢纽、大洞口水库等骨干水源工程前期工作扎实开展，新增解决15.67万农村人口饮水安全问题。完成电网建设投资4.09亿元。村村通宽带工程稳步实施。3G网络覆盖面进一步扩大。中缅油气管道建设工程顺利推进。

　　——我们全力加强产业园区建设，工业发展进一步加快。全年实现全部工业增加值114.46亿元，增长15.7%；500万元及以上工业增加值95.15亿元，增长21.6%。园区建设加快推进，管理体制逐步理顺，完成基础设施建设投资35亿元，建成标准化厂房50万平方米，新增入园企业63家，园区实现工业总产值171.6亿元，占全市工业总产值44.3%。工业项目建设进度加快，黄果树铝业二期、台泥水泥二期、港安水泥等项目建成投产，久联民爆、红星钡盐技改等项目加快推进，完成工业投资121.08亿元，增长43.3%。实施高载能企业用电补贴，补助资金5270万元。

　　——我们全力加快中心城区建设，城乡面貌进一步改变。中心城区详规覆盖率达90%，县城详规覆盖率达70%，编制完成乡镇规划22个、村庄规划400个。打击违法建设力度不断加大，“两违”蔓延势头得到初步遏制。中心城区路网等基础设施建设加快推进，北部新区和新火车站片区基础设施建设全面启动，二环路、市便民服务暨会展中心开工建设，中华西路建成通车。城区主干道“白改黑”和路灯改造全部完成，虹山湖景观整治一期工程顺利完工，若飞公园建成投用，“净化、绿化、亮化”工程成效明显，城市面貌焕然一新。各县城基础设施建设步伐加快，安普城市快速干道、关岭灞陵大道、紫云新城东西大道等项目顺利推进。小城镇建设“六个一”工程稳步实施。贵安新区路网规划建设扎实推进。城市管理不断加强，“四创”工作深入开展，在“整脏治乱”和“满意在贵州”年度考核中，我市名列全省第一。

　　——我们全力调整农业产业结构，农民收入进一步增加。全市农林牧渔业总产值85.21亿元，增长11.2%。粮食总产量65.15万吨，实现恢复性增长。种植业结构不断优化，蔬菜、茶叶、中药材、精品水果等特色优势农产品种植规模不断扩大，粮经作物种植比例由2011年的51∶49调整为44∶56。现代生态畜牧业加快发展，畜牧业产值占农林牧渔业总产值比重达47.3%，比2011年提高1.2个百分点。安顺全国丘陵山地农业机械化示范区启动建设，全市农机总动力达145万千瓦，增长8.1%。农业产业化水平进一步提高，龙头企业加快发展，农民专业合作社达513家。新农村建设扎实推进，完成村庄整治45个共1721户，“四在农家”创建活动广泛开展。黄果树和谐家园示范工程启动实施。

　　——我们全力推动旅游产业转型，第三产业进一步发展。黄果树旅游集团股权划转工作全面完成，龙宫、格凸河等景区引入市场主体进行深度开发，全市旅游文化资源逐步整合。旅游配套设施不断完善，百灵·希尔顿、国际佳缘等高星级酒店建设加快，一批商务酒店、连锁酒店和富有地方民族特色的农家乐蓬勃发展。安顺直飞北京、重庆等地航线开通运营，黄果树瀑布节、龙宫油菜花旅游节等大型节庆活动成功举办。全年接待游客2243.94万人次，增长28%，实现旅游收入195.04亿元，增长30%。金融保险、交通物流、邮电通信、商业零售、信息服务等现代服务业快速发展，第三产业增加值162.41亿元，增长16.5%。

——我们全力加强生态建设保护，生态环境进一步改善。水利建设、生态建设、石漠化治理“三位一体”规划深入实施。完成营造林34.64万亩，完成小流域治理项目6个，治理水土流失207平方公里，治理石漠化100平方公里，森林覆盖率达40.5%。强力推进节能减排，淘汰落后产能114万吨，单位生产总值能耗降低3.2%，化学需氧量、氨氮和二氧化硫排放总量分别下降8.1%、18.3%和14.7%。全市生活垃圾无害化处理率提高16.4个百分点，日处理生活污水能力达8.3万吨，集中式饮用水源全面达标，城市环境空气质量优良率保持100%。

　　——我们全力深化改革扩大开放，发展活力进一步增强。安顺多种经济成分共生繁荣试验区改革不断深化，民营经济发展壮大，全市民营经济增加值占生产总值的比重达53%。成立市国资委，进一步强化国有资产监督管理。农村商业银行组建工作顺利推进，村镇银行、小额贷款公司、融资性担保机构稳步发展。齐伯、乐平、大山等6个乡撤乡设镇。农村综合配套改革和财政管理体制、医药卫生体制、事业单位等改革稳步推进。开放水平明显提高，与对口帮扶城市和“长三角”、“珠三角”等地区的联系合作进一步加强。组织参加赴港招商、第二届中国（贵州）国际酒类博览会等重大招商引资活动，首次安排1000万元奖励企业纳税大户和招商引资工作先进单位。全年招商引资实际到位资金465.83亿元，其中引进省外境内资金289.97亿元，分别是2011年的2.3倍和2.2倍。

　　——我们全力保障改善民生，人民生活水平进一步提高。新增城镇就业3.17万人，其中登记设立微型企业2889户，带动就业1.8万人，城镇登记失业率3.2%。城乡基本养老保险、全民医保实现全覆盖，参保人数持续增加。城乡低保保障标准提高10%，认真实施农村低保季节性缺粮户粮食救助制度，对城乡低保家庭发放3222.25万元粮油实物补助，成立流浪妇女儿童救助中心，社会救助工作进一步加强。城镇保障性住房新开工2743套，竣工7774套，完成农村危房改造2.5万户。全年减少农村贫困人口15.37万人，6个贫困乡镇实现减贫“摘帽”。启动扶贫生态移民工程，12个安置点房屋主体工程基本完成。全力开展“保供稳价”工作，居民消费价格指数全年涨幅控制在3.0%以内。各级各类教育加快发展，学前教育、高中阶段教育毛入学率分别比2011年提高8.15和11.77个百分点，农村义务教育学生营养餐补助在原有3元标准基础上增加1元。科技事业取得新进展，顺利通过国家知识产权试点城市验收。文化事业和文化产业发展势头良好，苗族英雄史诗《亚鲁王》整理出版，一批文艺作品在全国全省大型赛事中取得优异成绩，广播电视村村通工程在全省率先完成。全民健身运动广泛开展，竞技体育水平不断提高。基层医疗服务能力明显提升，新型农村合作医疗参合率达98.06%，市人民医院异地新建工程加快推进。全市人口出生率12.39％。、自然增长率5.79％。，实现“双降”目标。加强和创新社会管理，成立群众工作部，强化社会矛盾纠纷排查疏导，畅通信访渠道，及时化解不稳定因素。安全生产监管体系不断完善，安全生产形势总体稳定。扎实推进“平安安顺”创建工作，群众安全感指数达89.35%，比2011年提高9.43个百分点。国家安全、国防教育、民族、宗教、双拥、老干、档案、史志、外事、侨务、对台、人防、气象等工作进一步加强。

　　一年来，我们加快转变政府职能，努力提高服务发展的能力。自觉接受人大及其常委会的法律监督和工作监督，自觉接受政协的民主监督，办理人大代表建议119件、政协委员提案193件，满意率100%。认真听取各民主党派、工商联和无党派人士的意见和建议，积极支持工会、共青团、妇联、科协、残联、红十字会等人民团体依法按章履行职责。简化办事程序，优化审批流程，提高办事效率，精简行政许可事项19项，下放26项，完善市、县两级政务服务中心建设，市公共资源交易中心投入运行。全面推行电子政务。应急管理工作进一步加强，预防处置突发公共事件的能力得到提高。加大反腐倡廉力度，加强行政监察和审计监督，切实做到勤政、廉洁、务实、高效。

　　各位代表！2012年我市经济社会发展取得的成绩，是省委、省政府和市委坚强领导，省直各部门大力支持的结果，是全市各族干部群众抢抓机遇、团结奋斗的结果。在此，我代表市人民政府，向全市各族人民，向人大代表、政协委员，向各民主党派、工商联、无党派人士和各人民团体，向驻安人民解放军、武警官兵、人民警察、中央和省驻安单位，向所有关心和支持我市改革开放和现代化建设的各界人士，表示衷心的感谢并致以崇高的敬意！

　　在肯定成绩的同时，我们也清醒地认识到，我市经济社会发展中还存在不少困难和问题，主要是：经济总量小、人均水平低，贫困人口多，全面小康建设任务十分艰巨；工业经济短板，产业结构单一；城镇建设滞后，辐射带动能力不强；体制机制还不健全，动力活力有待提高；公共服务能力不足，社会管理水平不高，改善民生、维护稳定的压力还很大；发展环境不优，行政效率较低，少数干部作风漂浮，与“十破十立”的差距很大。这些问题，我们一定认真对待、认真研究、认真解决。

　　二、2013年的重点工作

　　2013年是全面贯彻落实党的十八大精神的开局之年，是实施“十二五”规划承前启后的关键一年，是为与全省全国同步全面建成小康社会奠定坚实基础的重要一年，做好今年的各项工作，意义重大。

　　当前，我市发展机遇与挑战并存。一方面，国际形势复杂多变，国内经济社会发展中不可预见的矛盾和困难仍然较多；省内各市州不甘落后、争先图强、加快发展的态势逼人，对人才、技术、资本等生产要素的争夺更加激烈；全市经济社会发展面临土地、资金等要素制约，实现提速赶超的任务十分艰巨。另一方面，我们更要看到前所未有的发展机遇和有利条件：从国家层面看，党的十八大胜利召开，国家发展形势稳定，经济企稳运行。中央继续实施积极的财政政策和稳健的货币政策，国家新一轮西部大开发、新一轮扶贫开发、滇桂黔石漠化集中连片特殊困难地区扶贫攻坚工程深入实施，东部产业加快转移，为我市争取支持加快发展创造了有利条件。从省的层面看，黔中经济区规划建设上升为国家战略，贵安新区启动建设，全省发展驶入快车道，必将给我市提速发展带来更多机遇。从市的层面看，我们发展思路更加清晰，工作重点更加明确，工业化、城镇化带动战略初显成效，广大干部职工的精气神极大提振，干事创业、增比进位、赶超跨越的氛围日益浓厚，提速发展的内生动力更加强劲。

　　总的来看，机遇远大于挑战。我们要以党的十八大精神为指引，认真贯彻落实省委十一届二次全会、市委三届三次全会精神，坚持主基调、主战略，按照“稳中求进、进中求快、快中保好、更好更快”的总要求，紧紧围绕“提速发展、奋力赶超、同步小康”这一目标，全力抓好固定资产投资和招商引资两个关键，切实做好基础设施、生态环境、社会民生三个方面重点工作，大力推进工业化、城镇化、农业现代化和旅游产业化“四化”同步发展，加快推动信息化与“四化”相融合，努力实现全市经济社会又好又快、更好更快发展。

　　2013年，全市经济社会发展的主要目标是：地区生产总值增长18％；全社会固定资产投资660亿元，增长65％；招商引资实际到位资金769亿元，增长65%；公共财政预算收入增长25％；社会消费品零售总额增长18%；城镇居民人均可支配收入增长18%；农民人均纯收入增长20%；城镇登记失业率控制在4.2%以内；人口自然增长率控制在5.7％。以内；居民消费价格指数控制在省调控范围内；完成省下达的节能减排任务。

　　围绕实现上述目标，重点抓好以下九个方面工作:

　　（一）坚定不移扩大固定资产投资，着力抓好项目建设，促进经济发展新提高

　　切实提高项目谋划水平。加强项目库动态管理，围绕国家和省政策导向和重点支持方向，及时编报一批基础设施、产业发展、社会民生等方面的大项目、好项目，确保我市项目库投资规模保持在10000亿元以上。加大项目前期工作力度，提高项目成熟度，市级继续安排5000万元用于项目前期工作，切实保障重大工程和重点项目前期工作经费需求。全力抓好国发〔2012〕2号、黔党发〔2012〕15号文件的项目化、实物化、资金化、时限化落实，积极争取国家和省的支持，确保新开工一批重大工程和重点项目。

　　强力推进项目实施。认真抓好195个亿元以上重大工程和重点项目的责任分解，明确每个项目的牵头领导和责任单位。定期召开调度例会，切实加强项目调度，在工程进度、质量安全、资金保障、手续完善及入库统计上狠下功夫。强化项目跟踪落实，加大督促检查力度，严格执行问责制度。实行项目建设过程审计监督，防止项目单位随意超概算。依法严厉打击强揽工程、强买强卖、强拉强运、强行阻工等行为，营造良好的建设环境。

　　全力抓好项目要素保障。积极争取国家和省增加我市用地计划指标，加强土地开发复垦，清理闲置土地，用足用好土地增减挂钩、低丘缓坡开发利用试点政策，确保2000公顷项目建设用地。加大资金保障力度，大力实施“引金入安”工程，进一步加强“政银企”合作，确保贷款余额增长30%以上。完成安顺农村商业银行、紫云农村商业银行组建工作，新建村镇银行3家，规范发展小额贷款公司和融资性担保机构。充分发挥市国资公司、城投公司、交投公司、工投公司等投融资平台的作用，千方百计扩大融资规模。积极支持和帮助企业多渠道融资，鼓励企业上市融资和再融资。

　　（二）坚定不移实施工业强市战略，着力加快园区建设，推动工业经济新增长

　　加大工业投资力度。多渠道筹集工业发展资金，充分发挥好财政资金的杠杆作用，撬动更多社会资金投入工业发展，确保完成工业投资240亿元。加快工业项目建设进度，强力推进安电三期、久联民爆、华兴玻璃、顺立达纺织等一批重点项目建设，推动项目早日投产、达产。

　　增强园区聚集能力。集中力量重点打造10个省级产业园区，大力推进市直管园区建设。争取设立民用航空产业基地专项建设发展基金。推动工业园区差别化、特色化发展，积极发展园区主导产业和龙头企业，促进更多关联企业聚集发展。加快园区投融资平台建设，运用市场手段，多形式、多渠道筹集资金，着力完善园区生产性、生活性服务设施和环保设施，确保实现“五通一平”、努力实现“七通一平”，全年完成标准化厂房建设100万平方米。

　　提升工业生产运行水平。全力保障煤、电、油、运等生产要素供应，确保全年煤炭产量1600万吨、发电量90亿千瓦时。加大企业帮扶力度，帮助企业开拓市场，积极做好驻安军工企业对接服务工作，鼓励大企业集团和重点企业扩大生产规模，扶持中小企业做大做强。确保全部工业增加值138亿元，增长19%；500万元及以上工业增加值118亿元，增长25%。

　　提高企业创新能力。支持企业进行技术改造和设备更新，促进企业扩能增效。鼓励企业加大科研投入，与高校、科研院所深入协作，创新技术工艺，提高产品附加值。以煤矿企业为重点，推进同类或跨行业企业组建大企业大集团，提升企业综合经营管理水平和抗风险能力。培育发展科技服务中介机构，加快成果转化，提高知识产权的申请量和授权量，促进知识产权资本化、商品化。

　　（三）坚定不移实施城镇化带动战略，着力扩大城镇规模，促进辐射带动能力新提升

　　提高规划管理水平。认真抓好各级各类规划编制工作，中心城区详规覆盖率100%，各县城详规覆盖率85%以上，小城镇总规覆盖率100%。严厉打击违法建设和违法用地，确保拆违控违工作取得明显成效。加快推进数字化城市管理平台、“天网工程”、智能公交等系统建设，实现联网运行、信息共享。继续推进“净化、绿化、亮化”工程，全面开展道路交通、市场秩序、“前堂后灶”综合整治，深入开展“四创”工作，继续保持和扩大“整脏治乱”和“满意在安顺”工作成果，进一步提高人民群众满意度。

　　拉大城市框架。加快北部新区、新火车站片区基础设施建设，确保二环路年底全线贯通，推进北四号路、武当路二期等路网建设。加快市便民服务暨会展中心、市体育中心等重大项目建设，推进中华西路棚户区和汪家山、管元“城中村”改造，启动虹山湖景观整治二期工程和历史文化街区改造一期工程。加快普定、平坝、镇宁与中心城区同城化发展步伐，确保安普城市快速干道建成通车，开工建设贵黄城市快速干道。

　　推动产城互动发展。坚持以城带产，以产促城，统筹推进园区规划建设与城镇建设发展。高标准合理规划功能布局，重点打造10个城市综合体。大力推进县城新区开发和旧城改造，加快建设各县城主干道等基础设施。注重特色发展，重点打造13个省、市级示范小城镇，引导相关产业向小城镇集中布局，培育县域经济新的增长点。配合做好贵安新区直管区范围内征地拆迁等相关工作，用足用好用活贵安新区的优惠政策，加大市域内非直管区建设力度，借势加快发展。

　　提升综合承载力。加快推进污水收集管网、污水处理二期、生活垃圾收运系统等工程建设。优先发展公共交通，建成市公交枢纽总站并投入运营，增加和调整优化城市公交运营线路，公交车、出租车逐步推广使用天然气。完善交通指挥系统，有效缓解中心城区交通拥堵状况。加快天然气门站及环城高压管网建设进度，开工建设中心城区燃气二期工程，扎实推进各县城燃气管道建设。

　　（四）坚定不移推进农业现代化，着力优化农业产业结构，增强农村发展新活力

　　夯实农业农村发展基础。认真落实各项强农惠农政策，完善财政支农资金稳定增长机制。继续推进安顺全国丘陵山地农业机械化示范区建设。抓好高标准农田建设和中低产田土改造。增强农业科技创新和成果转化能力，提高科技对农业增长的贡献率。发挥山地特色和比较优势，重点打造6个以上省级现代高效农业示范园区。加强农民职业技能培训，促进就地就近就业，增加工资性收入。抓好“整乡推进”新农村建设工作，继续开展“四在农家”创建活动。加快推进黄果树和谐家园示范工程建设。启动实施顶云新区城乡统筹发展示范工程。

　　优化农业产业结构。稳定粮食生产，提高单产水平，确保粮食种植面积212万亩。加大种植业结构调整力度，种植蔬菜106万亩，新增茶园6.5万亩、中药材13万亩、水果15.5万亩（其中金刺梨7万亩），收购烤烟27万担，粮经作物种植比例调整到40∶60。加快发展现代生态畜牧业，加大地方畜禽品种保护力度，实现肉蛋总产量16.6万吨，水产品产量1.2万吨，牧渔业产值增长10%，占农林牧渔业总产值的比重提高1个百分点以上。

　　推动农业产业化发展。着力构建集约化、专业化、组织化、社会化相结合的新型农村经营体系。加大龙头企业扶持力度，引进农产品精深加工企业，支持牛来香、百花串等农特产品做大做强。大力支持和引导农民专业合作社加快发展，努力提高农民的组织化程度。建立健全农产品质量监管机制，做好“三品一标”工作，积极推进农产品质量安全标准示范县建设。完善农技推广、市场信息和动植物疫病防控体系。

　　（五）坚定不移推进旅游产业化，着力发挥旅游业龙头作用，促进第三产业新发展

　　加强旅游景区品质品牌建设。重点打造7个旅游景区，建设以“黄果树—龙宫度假旅游综合体”为核心，辐射格凸河、屯堡、关岭古生物国家地质公园、夜郎湖、坝陵河、多彩文化万象旅游城等景区的泛黄果树旅游圈。加强旅游景区基础设施建设，提高便利性和舒适性，加快推进黄果树、龙宫景区总体规划修编和深度开发。加强旅游景区综合管理和功能配套，提高服务质量和管理水平，推进“智慧旅游”系统建设，加快百灵·希尔顿、国际佳缘、华荣、葡华等高星级酒店建设进度。加强旅游景区整体包装、对外宣传和市场营销，进一步提高知名度和影响力，确保全年接待游客2918万人次以上，增长30%，旅游总收入255亿元以上，增长30%。

　　促进旅游产业优化升级。深入挖掘文化内涵，推进旅游与文化、旅游与体育融合发展，创新旅游业态和转变发展方式，提高旅游产业竞争力和带动力。加快推进黄果树旅游集团公司上市步伐。吸引有实力的旅游企业落户我市，增强旅游市场活力。制定安顺旅游形象标识。创新方式办好黄果树瀑布节、油菜花旅游节等节庆活动。举办坝陵河低空跳伞、格凸河攀岩、镇宁半程马拉松等国际体育赛事。打造一台高水准的大型实景演出，编排一批室内演出剧目。

　　大力发展现代服务业。加快规划建设黄桶幺铺现代物流园区。抓好汽车、建材、小商品等专业市场建设。加大规范化农贸市场建设力度，建成西秀产业园区农产品批发市场，推进黔中农产品交易大市场建设。采取灵活的政策措施，促进房地产业健康发展。大力发展信息服务、现代金融、交通运输等生产性服务业，加快发展餐饮、住宿、家庭服务、社会养老等生活性服务业。

　　（六）坚定不移加强基础设施建设，着力完善交通水利设施，促进发展条件新改善

　　推进交通基础设施建设。配合做好长昆快速铁路和惠兴、六镇、厦蓉、织普等高速公路建设工作，全面开工建设安紫高速公路，确保安木公路年内建成通车，启动实施镇宁至乐纪公路改扩建工作，大力推进通村油路（水泥路）建设。加大场站建设力度，开工建设安顺客运东站，加快紫云等县城交通枢纽中心建设。大力培育新航线，争取开通安顺至上海、广州等地航班。

　　推进水利工程建设。配合做好黔中水利枢纽工程建设工作，完成黄家湾水利枢纽工程项目前期工作，全面建成“引千入虹”工程，加快推进安西灌区和石朱桥、新场河、三岔河、纳井田等水库工程，开工建设大洞口水库和偏坡水库，完成中小河流治理工程10个，治理病险水库15座。

　　推进电力、信息等基础设施建设。加强城乡电网建设与改造，切实增强产业园区供电保障能力，完成电网投资3.2亿元。加快“数字安顺”建设，有序推进信息化与工业化、城镇化、农业现代化、旅游产业化深度融合，加快宽带提速、3G网络改造等工程建设，实施数字安顺地理空间框架建设国家试点计划项目。配合做好中国移动全国数据中心和江苏慧谷产业园区建设。

　　（七）坚定不移加强生态文明建设，着力加大自然生态系统和环境保护力度，推进美丽安顺建设取得新进展

　　加强生态工程建设。实施退耕还林、天然林保护二期、珠江防护林体系建设等重点林业生态工程，完成营造林35万亩，确保森林覆盖率42%以上。治理水土流失150平方公里，完成石漠化综合治理67平方公里。加强科技防灾减灾体系建设，增强城乡防洪抗旱排涝能力，提高气象、地质、地震灾害防御能力。

　　大力推进节能减排。落实重点企业、行业和区域的节能减排责任，淘汰一批落后产能，引导和支持企业加强节能技改，调整产品结构，降低能耗和生产成本。削减污染物排放总量，确保氨氮、二氧化硫等主要污染物排放总量控制在国家规定范围内。推广应用节能建筑材料和高效节能环保产品。

　　加强环境保护。坚持从开发建设的源头控制新的环境污染和生态破坏。加强“三废”治理和综合利用。强化集中式饮用水源地环境保护，确保水质全面达标。加强城市景观水环境综合治理，努力改善水环境质量。加强农业面源污染治理，以创建生态示范乡、村为抓手，着力推进农村环境综合整治。实施地质灾害和矿山环境恢复治理工程。加强生态文明宣传教育，增强全民环保意识，营造爱护生态环境的良好风气。

　　（八）坚定不移深化改革扩大开放，着力加强招商引资，推动对外开放水平迈上新台阶

　　深入推进各项改革。继续深化安顺试验区改革，弘扬“敢闯敢试、敢为人先”的精神，寻求试验区跨越发展的新载体，探索以开放促开发、开发促发展的新途径，赋予试验区改革的新内涵。大力发展民营经济，新增规模以上民营企业50家，民营经济增加值占生产总值的比重达到60%。完善国有资产管理体制，促进国有资产保值增值。继续推进扩权强县，促进县域经济加快发展。深化财税体制改革，提高财政资金使用效益。积极稳妥推进基层管理体制改革，夯实基层基础，抓好乡改镇、镇改办、村改居工作，加快户籍制度改革步伐。深入推进农村综合配套改革和国有企业、事业单位等各项改革。

　　提高开放型经济发展水平。创新招商引资方式，大力开展驻点招商、以商招商，鼓励全民招商，形成“大招商”格局。积极参加第九届“泛珠会”、第三届中国（贵州）国际酒类博览会、贵州·香港投资贸易活动周等大型招商引资活动。加强与对口帮扶城市、友好城市及经济发达地区的合作。积极推进外向型经济发展，加快外贸基地建设，重点培育一批出口加工企业，鼓励优势产品扩大出口。

　　打造良好投资环境。进一步强化政务服务中心、公共资源交易中心的作用。加强协调服务，建立健全从项目立项、审批到投产达产的全程跟踪服务机制，提高履约率、资金到位率、开工率、投产达产率。加强投资环境测评工作，加大对影响投资环境行为的查处力度，为企业提供全面、周到、细致的服务，努力营造亲商、敬商、爱商的良好环境，吸引各类投资者到我市投资兴业。

　　（九）坚定不移推进社会事业发展，着力提高人民群众幸福指数，开创和谐稳定新局面

　　提升公共服务水平。进一步落实促进就业的各项政策措施，拓宽就业渠道，帮助就业困难人员就业。继续实施“3个15万元”扶持政策，新增微型企业2500户。加快完善覆盖城乡的社会保障体系建设，提高保障水平。继续实施农村低保季节性缺粮户粮食救助制度。积极探索医疗救助新机制，扩大救助覆盖面。推进保障性安居工程建设，改善低收入家庭基本住房条件。坚持教育优先发展，压缩行政经费的5%用于发展教育，统筹建立教育发展基金，加快推进“四项突破工程”，启动实施“9+3”义务教育及三年免费中职教育计划，开工建设安顺职业技术学院新校区，扶持发展民办教育。加强专业技术人才队伍建设，做好重点领域、重点产业、重点学科、重点项目急需人才的引进调配工作。深入推进文化体制改革，加快发展文化事业和文化产业，启动市人民影剧院、图书馆等项目建设，实施“六个一批”文化工程。积极推进医药卫生体制改革，抓好重大传染病、地方病防治工作，提高医疗卫生保障服务水平。统筹抓好人口和计划生育工作，确保实现“双降”目标。

　　加强和创新社会管理。学习借鉴“贵阳经验”、“铜仁经验”、“余庆经验”，大力加强社区建设管理、社会风险评估、基层矛盾化解工作。推广“阳光工程”，建立社区戒毒康复新模式。实施“三关爱”工程。加强对流浪儿童、乞讨人员救助管理。依法保障劳动者合法权益，坚决防止年终讨薪现象发生。强化以煤矿为重点的安全生产基层基础建设，狠抓安全生产各项责任制的落实，实现安全生产事故起数和死亡人数“双降”。强化食品药品安全监管。完善突发公共事件应急管理机制，切实加强应急处置能力建设。继续深入开展“平安安顺”创建活动，抓好城市报警和监控系统建设，强化治安防控，提高见警率，加大对各种违法犯罪的打击力度，进一步提高人民群众的安全感。支持国防、军队建设和法院、检察院工作。加强国家安全、司法行政、双拥、民族、宗教、外事、侨务、对台、人防、档案、史志、气象等工作。

　　切实办好“十件实事”。2013年市委、市政府决定为民办“十件实事”：⑴建设600公里通村油路（水泥路），覆盖全市8个县区、180个村寨；⑵完成2万人扶贫生态移民搬迁，新开工建设保障性住房2万套，改造农村危房1.7万户；⑶完成广播电视“户户通”5.6万户建设项目；⑷完成农村20万人安全饮水工程，完成安顺市中心城区供水二期工程；⑸减少农村扶贫对象10万人；⑹城乡最低生活保障标准在2012年基础上提高10%以上；⑺全市完成城镇新增就业42000人，新增创业户1200户，创业带动就业4800人；⑻完成113所农村中小学食堂标准化建设、43所农村中小学学生宿舍建设，完成33所乡镇公办幼儿园建设，实现乡镇公办幼儿园全覆盖；⑼实现新型农村合作医疗全覆盖，确保参合率稳定在95%以上，加快推进市人民医院整体搬迁和市儿童医院项目建设，年内完成2所医院门诊大楼主体工程；⑽切实解决市区交通拥挤和广大市民出行难、停车难问题，年内在市区新增150辆出租车、2000个公共停车位，新建5座人行天桥。通过“十件实事”的完成，实现好、维护好、发展好人民群众的根本利益，使改革发展成果惠及广大人民群众！

　　三、切实加强政府自身建设

　　与全省全国同步全面建成小康社会的宏伟目标对政府工作提出了更高要求，我们必须进一步解放思想，开拓创新，努力建设学习型、服务型、创新型、法治型、廉洁型政府。

　　（一）提高素质，与时俱进，建设学习型政府。坚持把学习作为一种本领、一种常态、一种生活方式，提高对新情况、新事物的认识水平，增强谋全局、抓机遇、解难题、求突破的能力。立足安顺实际，完善干部学习制度，采取多种形式，丰富学习内容，促进各级干部解放思想、拓展思路、开阔视野、提升境界，把学习的着眼点放到研究和解决实际问题上，使学习的成果体现到工作成效上。

　　（二）转变作风，提高效率，建设服务型政府。加快行政审批改革，加强效能建设，提高行政效率，解决拖、懒、散等问题，改变“上下急、中间拖”的“中梗阻”现象。按照精致、细致、极致的要求，不断提高工作执行力和落实力。进一步加大对乱作为、不作为、不敢作为、不善作为等行为的整治力度。严格执行中央、省委和市委关于改进工作作风密切联系群众的规定，讲实话、干实事，真心实意为人民群众搞好服务。

　　（三）解放思想，深化改革，建设创新型政府。坚持“十破十立”，把改革创新贯穿于政府工作的全过程。创新经济工作方式，遵循发展规律，勇于先行先试，自觉运用市场经济的手段和方法推动经济工作，探索具有安顺特色的发展模式。创新决策方式，建立重大决策协商和征求意见制度，健全重大事项咨询、公示、听证制度，提高决策的透明度，促进决策科学化、民主化。创新工作方法，坚持不懈抓重点、攻难点、增亮点，善于总结经验，以点带面，推动全局。

　　（四）依法行政，接受监督，建设法治型政府。牢固树立依法治市理念，严格依法行政，强化行政执法，切实做到严格、规范、公正、文明。深入实施“六五”普法，提高全社会法律意识。加强行政复议和行政调解，深入推进政务公开和政府信息公开，不断扩大公开内容和范围，推行网络政务。自觉接受人大、政协、人民群众和新闻媒体的监督，认真办理人大代表建议和政协委员提案，积极听取各民主党派、工商联、无党派人士和工、青、妇等人民团体的意见建议。

　　（五）勤政廉政，率先垂范，建设廉洁型政府。按照“干部清正、政府清廉、政治清明”的要求，注重反腐倡廉教育，自觉遵守廉政准则。加强惩治和预防腐败体系建设，重视行政监察和审计监督，严惩腐败分子。坚决纠正损害群众利益的不正之风，切实解决群众反映强烈的突出问题。强化预算约束和财政管理，严格各项支出，厉行勤俭节约、反对铺张浪费，努力建设人民群众信赖拥护的廉洁型政府。

　　各位代表！事业凝聚人心，团结昭示力量，行动铸就辉煌！站在新的起点，让我们更加紧密地团结在以习近平同志为核心的党中央周围，深入贯彻落实党的十八大精神，在市委的坚强领导下，紧紧依靠全市各族人民，以更加饱满的热情，更加昂扬的斗志，更加务实的作风，解放思想，抢抓机遇，凝聚力量，攻坚克难，向着全面建成小康社会的宏伟目标阔步前进！
